# COMPARISON OF THE PREDICTIVE VALUE OF UPPER LIMB SOMATOSENSORY EVOKED POTENTIALS AND MOTOR EVOKED POTENTIALS FOR FUNCTIONAL RECOVERY IN SUBACUTE STROKE: A RETROSPECTIVE STUDY

**DOI:** 10.2340/jrm.v58.45010

**Published:** 2026-03-10

**Authors:** Jungwoo SHIM, Changju KIM

**Affiliations:** 1 Department of Rehabilitation Medicine, Chungnam National University Sejong Hospital, Sejong-si; 2 Department of Physical Therapy, Cheongju University, Cheongju-si, Republic of Korea

**Keywords:** activities of daily living, motor evoked potentials, somatosensory evoked potentials, stroke, upper extremity

## Abstract

**Objective:**

To compare the prognostic value of somatosensory evoked potentials and motor evoked potentials for upper limb functional recovery in patients with subacute stroke.

**Design:**

Retrospective observational analysis.

**Subjects/Patients:**

A total of 111 inpatients with subacute stroke who underwent upper limb somatosensory evoked potentials and motor evoked potentials testing within 1 week of admission and completed a standardized rehabilitation programme.

**Methods:**

Somatosensory evoked potentials and motor evoked potentials were categorized as non-responsive, abnormal, or normal. Discharge outcomes included the Fugl-Meyer Assessment for upper limb, Box and Block Test, Functional Independence Measure, and Korean version of the modified Barthel Index. Mixed-effects models were applied to examine associations between somatosensory evoked potentials or motor evoked potentials status and discharge outcomes, adjusting for baseline score and admission duration, with patient ID as a random intercept. Effect sizes were calculated using Cohen’s *f* ^2^***.***

**Results:**

Normal somatosensory evoked potentials were associated with higher Functional Independence Measure and Korean version of the modified Barthel Index scores than non-responsive somatosensory evoked potentials, while abnormal somatosensory evoked potentials showed non-significant trends. For motor evoked potentials, the normal group showed higher Box and Block Test scores, and both abnormal and normal groups had higher Korean modified Barthel Index scores than non-responsive.

**Conclusion:**

Admission somatosensory evoked potentials and motor evoked potentials provide complementary prognostic information in subacute stroke rehabilitation.

## LAY ABSTRACT

Recovery after stroke varies widely among patients, and predicting who will regain good arm and hand function remains challenging. This study examined whether 2 nerve tests, somatosensory evoked potentials and motor evoked potentials, can help predict recovery in people with subacute stroke. We reviewed data from 111 patients who received both tests and completed rehabilitation. Somatosensory evoked potentials and motor evoked potentials results were grouped as non-responsive, abnormal, or normal, and their relationships with movement and daily function at discharge were analysed. Patients with normal somatosensory evoked potentials showed greater independence in daily activities, while those with normal motor evoked potentials performed better in hand coordination tasks. These findings suggest that somatosensory evoked potentials and motor evoked potentials reflect different aspects of recovery, and when combined may improve early prediction of functional outcomes. This information could help clinicians plan individualized rehabilitation programmes more effectively for patients after stroke.

Stroke is a leading neurological disorder that causes physical disabilities such as hemiparesis, significantly impacting patients’ quality of life and their ability to perform activities of daily living (ADL) (1). Among stroke-related impairments, upper limb dysfunction is particularly prevalent, affecting more than 70% of stroke survivors. Furthermore, over 60% of these individuals experience diminished hand dexterity (2). Because the upper limb plays a crucial role in both fine motor control and gross movement coordination, upper limb paralysis is considered one of the most fundamental and disabling consequences of stroke, often limiting a patient’s ability to return to independent living and social participation (3).

In clinical rehabilitation, accurately predicting the potential for upper limb recovery is critical for developing effective treatment plans. Early identification of prognosis enables clinicians to design personalized intervention strategies that match the patient’s functional level, ultimately improving both the efficiency and efficacy of rehabilitation (4). Among various prognostic tools, somatosensory evoked potentials (SEP) and motor evoked potentials (MEP) are neurophysiological assessments that have gained attention for their ability to objectively quantify the extent of neural pathway damage and predict recovery potential (5).

SEP evaluates the functional integrity of sensory pathways by recording cortical responses to peripheral nerve stimulation (6). In contrast, MEP assesses the excitability and continuity of the motor pathway by measuring responses to transcranial magnetic stimulation delivered to the motor cortex (7). Previous studies have reported that patients with preserved MEP tend to achieve greater upper limb recovery compared with those without detectable responses (8), and such evidence has been incorporated into structured prediction algorithms such as the predict recovery potential (PREP) algorithm and its updated version, PREP2 (9, 10). Recent evidence further supports the prognostic value of MEP in stroke recovery. Daghsen et al. (11) demonstrated that corticospinal tract integrity assessed by MEP was significantly associated with upper limb functional outcomes in subacute stroke, reinforcing the clinical relevance of MEP-based prognostication. However, these algorithms focus primarily on corticospinal tract integrity and do not consider sensory pathways, which are also essential for functional independence. Emerging evidence suggests that sensory impairment may independently influence motor recovery after stroke. Van Ravestyn et al. (12) reported that somatosensory deficits were associated with poorer upper limb motor outcomes, highlighting the importance of integrating sensory pathway assessment into prognostic evaluations.

Importantly, upper limb function contributes not only to motor recovery but also to independence in ADL, particularly in self-care and domestic activities. Because ADL measures such as the Functional Independence Measure (FIM) and Barthel index capture both motor and global independence, examining SEP and MEP in relation to these outcomes provides clinically meaningful insight into how neurophysiological integrity translates into everyday functioning (13). In line with this perspective, sensory-based therapeutic approaches have been shown to enhance motor and functional recovery after stroke, suggesting that preservation of sensory pathways may have direct clinical implications for rehabilitation planning (14).

Therefore, this study aimed to investigate the predictive value of upper limb SEP and MEP status at the time of admission for upper limb function and ADL performance on discharge in patients with subacute stroke. In addition, we compared the relative predictive strength of SEP and MEP to identify which modality offers more clinically meaningful information for use in rehabilitation settings.

## METHODS

### Study design

This retrospective observational study aimed to evaluate the predictive value of neurophysiological markers, specifically upper limb SEP and MEP, for functional recovery in patients with subacute stroke. Clinical data were retrospectively obtained from the electronic medical records of patients admitted to C Hospital in Sejong City, South Korea, between July 2020 and April 2023. All patients underwent SEP and MEP assessments, as well as baseline functional evaluations, at the time of admission. Follow-up functional assessments were conducted at the time of discharge. The duration of hospitalization varied according to each patient’s clinical condition and individual progress during rehabilitation.

### Participants

A total of 211 patients were initially screened for eligibility. Of these, 111 patients who met the inclusion criteria were included in the final analysis. The inclusion criteria were as follows: individuals aged between 18 and 80 years; diagnosis of hemiparesis resulting from subacute stroke; completion of upper limb SEP and MEP evaluations within 3 months of stroke onset; and availability of functional assessment data on both admission and discharge.

Patients were excluded if they had a history of bilateral or recurrent stroke, demonstrated cognitive impairment defined as a score below 10 on the Korean Mini-Mental State Examination (K-MMSE), had orthopaedic or progressive neurological conditions that could affect motor function, or had incomplete clinical or assessment records. This study was conducted as a retrospective secondary analysis using data originally collected under institutional ethical approval. The study protocol was approved by the Institutional Review Board of Sejong Chungnam National University Hospital (IRB No. 2023-05-008), and the requirement for informed consent was waived due to the use of de-identified data.

### Neurological assessments

**Fig. 1:**
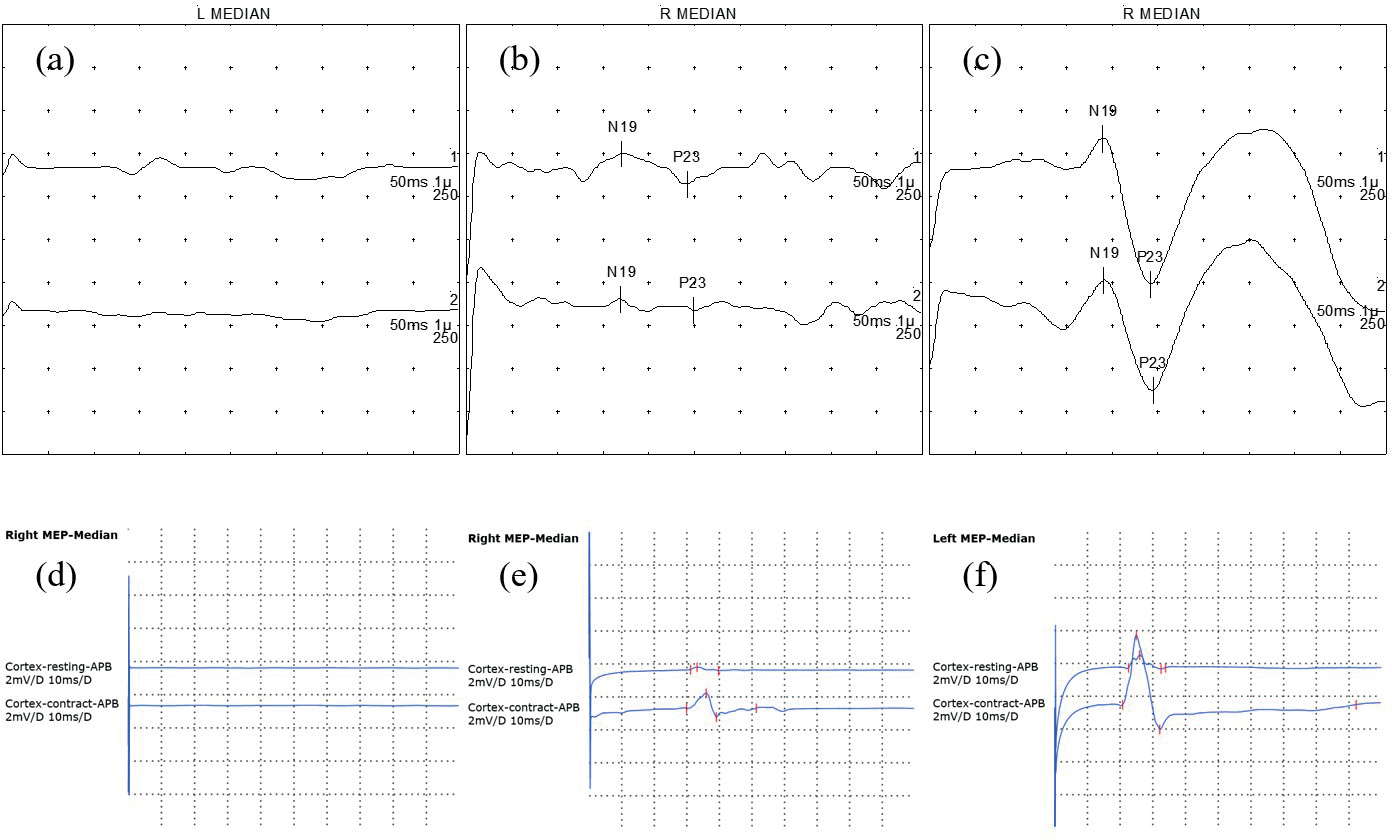
Representative examples of somatosensory evoked potentials and motor evoked potentials classifications. The horizontal axes represent time (ms), and the vertical axes represent response amplitude (µV) for both SEP and MEP waveforms. (A–C) Somatosensory evoked potentials waveforms: (A) non-responsive, no clear N19/P23; (B) abnormal, delayed or reduced N19–P23; (C) normal, preserved N19–P23 within normal limits. (D–F) Motor evoked potentials waveforms from the abductor pollicis brevis muscle: (D) non-responsive, no motor response; (E) abnormal, small, or inconsistent response; (F) normal, reliable response > 50 µV.

SEP testing was performed using an electrodiagnostic system (Electro Synergy System; Viasys Healthcare, Conshohocken, PA, USA, 2010). Electrical stimulation was applied to the median nerve at the wrist on the affected side, using an intensity of 20–30 mA and a frequency of 2–5 Hz (typically 3 Hz), in accordance with recommended guidelines (15). Cortical responses were recorded from Cz (active) and Fz (reference) electrodes following the international 10–20 system. Although multi-channel montages are recommended, a Cz–Fz derivation was used as per laboratory routine. A minimum of 250 responses were averaged to enhance the signal-to-noise ratio. An experienced neurologist visually classified SEP responses into 3 categories based on the N19 cortical waveform (equivalent to the N20 component described in international guidelines). Responses were considered normal when the N19 waveform was clearly identifiable, with a latency of 21.5 ms or less and preserved amplitude. Responses were classified as abnormal when the N19 latency exceeded 21.5 ms or when the cortical amplitude was markedly reduced. SEP was classified as non-responsive when no discernible cortical waveform was observed despite adequate stimulation (Fig. 1) (16). This 3-level classification was used as the primary independent variable in the statistical analyses.

MEP testing was conducted using a magnetic stimulator (MagPro X100; MagVenture, Farum Denmark, 2009) with a 126-mm parabolic encircling coil (MMC-140-II). Stimulation was applied over the contralesional motor cortex, and responses were recorded from the abductor pollicis brevis (APB) muscle on the affected side (17). The motor hotspot for the APB muscle was defined as the scalp position eliciting the largest and most consistent MEP amplitude in the relaxed muscle. Stimulus intensity was gradually increased up to maximal stimulator output when necessary to ensure that absent responses were not due to insufficient stimulation (18). An MEP was considered present when a reproducible peak-to-peak amplitude greater than 50 µV was elicited in at least 5 out of 10 consecutive trials (19). Based on waveform presence and relative amplitude characteristics, MEP responses were classified into 3 categories. Responses were classified as normal when reproducible MEPs with preserved amplitude and consistent morphology were observed. Responses were classified as abnormal when MEPs were present but showed markedly reduced amplitude compared with laboratory reference values or inconsistent morphology across trials. MEPs were classified as non-responsive when no discernible response could be elicited despite maximal stimulation (8, 9) (see Fig. 1). This categorical classification was used as the primary independent variable in the statistical analyses.

### Functional outcome measures

Functional recovery was assessed across 2 domains: upper limb motor function and ADL.

Upper limb motor function was evaluated using the Fugl-Meyer Assessment for upper extremity (FMA-U/L) and the Box and Block Test (BBT). The FMA-U/L quantitatively measures voluntary motor control of the shoulder, elbow, wrist, and hand through a series of items scored on a 3-point ordinal scale. It evaluates aspects such as joint movement, coordination, reflex activity, and synergy patterns. This tool has shown excellent inter-rater reliability, with intraclass correlation coefficients (ICC) as high as 0.98 (20).

The BBT was used to assess gross manual dexterity by asking participants to move as many wooden blocks as possible from 1 compartment to another using 1 hand over a 1-min period. The test is widely used due to its simplicity and sensitivity to change, and it demonstrates high test–retest reliability, with ICCs commonly reported above 0.91 (21).

ADL performance was measured using 2 standardized scales: the FIM and the Korean version of the Modified Barthel Index (K-MBI). The FIM consists of 18 items covering essential daily activities including self-care, mobility, communication, and social cognition. Each item is scored on a 7-point scale to indicate the level of assistance required (22).

The K-MBI assesses 10 basic ADL tasks, such as feeding, grooming, toileting, ambulation, and stair climbing, with each item scored from 0 to 15, producing a maximum score of 100. Higher scores on both measures indicate greater functional independence. Both instruments have been extensively validated and exhibit strong inter-rater reliability, with reported ICCs of 0.95 for the FIM and 0.89 for the K-MBI (23).

These tools are widely utilized in both clinical and research settings to objectively monitor stroke recovery in motor and functional domains.

### Statistical analysis

All statistical analyses were performed using IBM SPSS Statistics version 25.0 (IBM Corp, Armonk, NY, USA), with statistical significance set at *p* < 0.05.

First, descriptive statistics and frequency analyses were conducted to summarize the demographic and clinical characteristics of the participants. Continuous variables were reported as means and standard deviations, while categorical variables were presented as frequencies and percentages.

Second, correlation analyses were conducted to examine whether SEP or MEP status on admission was associated with functional outcomes on discharge. Normality of the continuous outcome variables was evaluated using the Shapiro–Wilk test. Based on the distribution, either Pearson’s correlation (for normally distributed variables) or Spearman’s rank correlation (for non-normally distributed variables) was applied. Although the analysis was correlational in nature, the use of baseline neurophysiological status and discharge outcomes allowed this step to serve as an initial assessment of predictive association.

Third, linear mixed-effects models were constructed to further assess the predictive value of SEP and MEP for functional recovery. SEP or MEP status on admission was entered as the fixed effect, and the corresponding discharge score for each outcome measure (FMA-U/L, BBT, FIM, K-MBI) was used as the dependent variable. Baseline functional score and length of hospital stay were included as covariates to adjust for initial condition and rehabilitation exposure. Patient ID was modelled as a random intercept to account for between-subject variability. This approach enabled the evaluation of the unique contribution of neurological status to recovery trajectories over time.

Effect sizes were calculated using Cohen’s *f*^2^, derived from the change in marginal *R²* between the full model (including SEP or MEP) and a reduced model (excluding SEP or MEP):


$$f^{2}=\frac{R_{\text{full}}^{2}-R_{\text{reduced}}^{2}}{1-R_{\text{full}}^{2}},R^{2}=\frac{V_{\text{null}}-V_{\text{full}}}{V_{\text{null}}}$$

In this formula, V represents the total variance of the model, including both residual variance and variance attributable to random effects. Thus, *f*^2^ reflects the proportion of additional variance in discharge outcomes explained by SEP or MEP status beyond that accounted for by baseline function and hospitalization duration.

## RESULTS

**Fig. 2:**
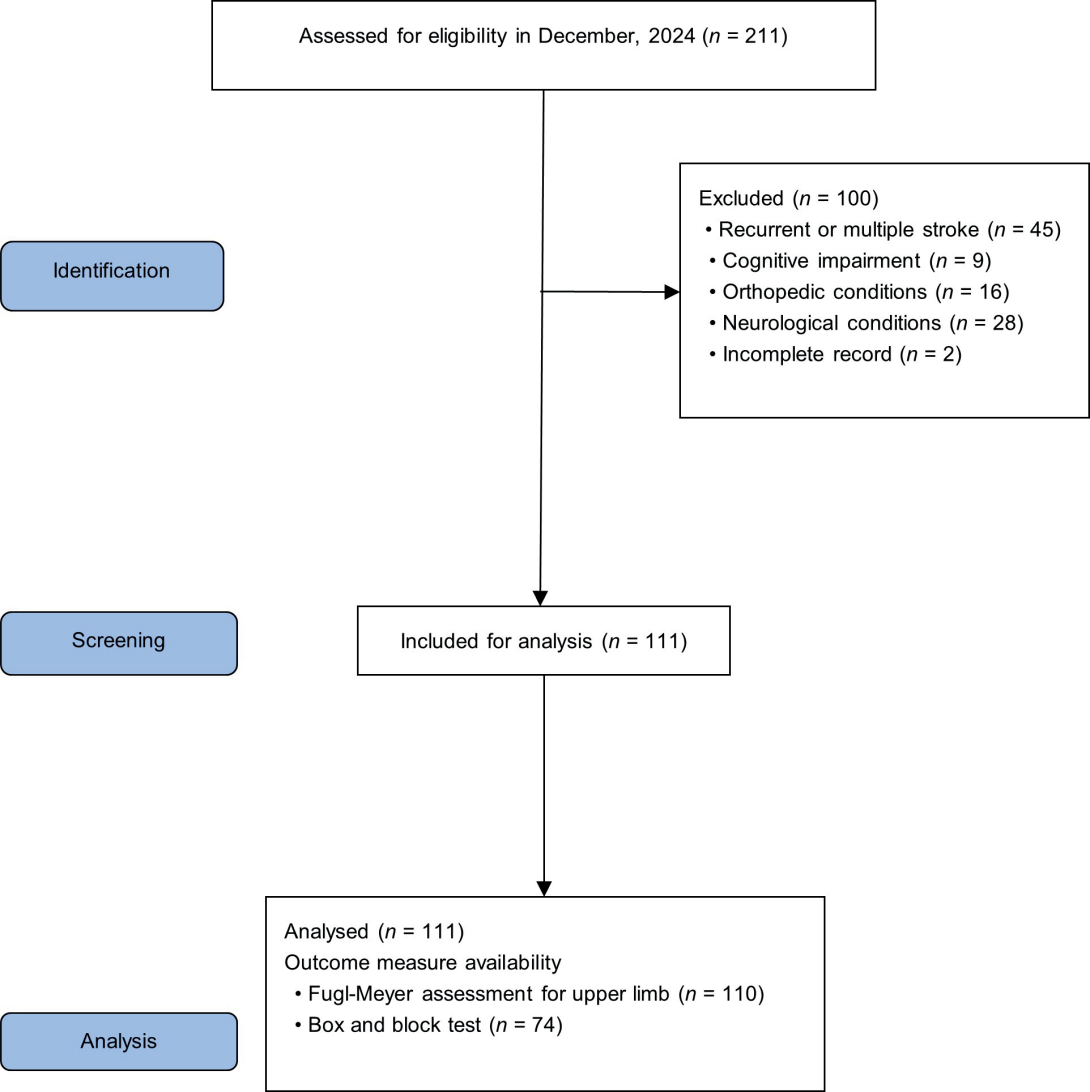
Flow diagram of data collection.

A total of 211 patients with subacute stroke were screened for eligibility. After applying the inclusion and exclusion criteria, 111 patients were included in the final analysis. The flow of patient screening and selection is illustrated in Fig. 2.

### General characteristics of subject

A total of 111 patients with subacute stroke were included in the analysis (Table I). The mean age was 64.55 ± 13.78 years, with a slightly higher proportion of male (61.3%) compared with female patients (38.7%). Regarding the affected side, 55.9% had left hemiparesis and 44.1% had right-sided involvement. Cognitive function, assessed using the K-MMSE, showed a median score of 23 (interquartile range [IQR], 15–26). The median duration of hospital admission was 21 days (IQR, 14–28), and the median time from stroke onset to the baseline functional assessment was 9 days (IQR, 7–10).

**Table I:** General characteristics and initial functional assessments of participants (*n* = 111)

Variables	Values
General characteristics	
Sex – male/female, *n* (%)	68 (61.3)/43 (38.7)
Affected side – left/right, *n* (%)	62 (55.9)/49 (44.1)
Age, years, mean ± SD	64.55 ± 13.78
K-MMSE, score, median (IQR)	23 (15–26)
Admission duration, days, median (IQR)	21 (14–28)
Time from stroke onset to baseline functional assessment, days, median (IQR)	9 (7–10)
Admission neurophysiological assessments	
Upper limb SEP status	
Non-responsive, *n* (%)	17 (15.3)
Abnormal, *n* (%)	55 (49.5)
Normal, *n* (%)	39 (35.1)
Upper limb MEP status	
Non-responsive, *n* (%)	37 (33.3)
Abnormal, *n* (%)	43 (38.7)
Normal, *n* (%)	31 (27.9)
Baseline functional scores at initial assessment, median (IQR)	
FMA-U/L (score) (*n =* 110)	66 (54–66)
BBT (*n =* 74)	27 (9–41)
FIM (score) (*n =* 111)	75 (54–91)
K-MBI (score) (*n =* 111)	66 (36–84)
Discharge functional assessment, median (IQR)	
FMA-U/L (score) (*n =* 110)	66 (64–66)
BBT (*n =* 74)	43 (24–53)
FIM (score) (*n =* 111)	94 (73–103)
K-MBI (score) (*n =* 111)	86 (71–93)

SD: standard deviation; IQR; interquartile range; K-MMSE: Korean Mini-Mental State Examination; SEP: somatosensory evoked potentials; MEP: motor evoked potentials; FMA-U/L: Fugl-Meyer Assessment for upper limb; BBT: Box and Block Test; FIM: Functional Independence Measure; K-MBI: Korean version of the modified Barthel Index.

Neurophysiological evaluations on admission revealed that, among the 111 patients, upper limb SEP responses were classified as non-responsive in 15.3%, abnormal in 49.5%, and normal in 35.1%. For upper limb MEP status, 33.3% were non-responsive, 38.7% were abnormal, and 27.9% showed normal responses.

Baseline functional assessments indicated a median FMA-U/L of 66 (IQR, 54–66) among 110 patients. The median BBT score was 27 (IQR, 9–41) in the 74 patients for whom data were available. For activities of daily living, the median FIM score was 75 (IQR, 54–91), and the median K-MBI score was 66 (IQR, 36–84).

On discharge, functional outcomes showed improvement across all measures. The median FMA-U/L score remained at 66 (IQR, 64–66), while the median BBT score increased to 43 (IQR, 24–53). The median FIM score on discharge was 94 (IQR, 73–103), and the median K-MBI score was 86 (IQR, 71–93).

### Association between baseline SEP and MEP status and functional outcomes on discharge

Correlation analyses were conducted to examine the association between upper limb SEP and MEP status on admission and functional performance on discharge. As indicated in Table II, upper limb SEP status was significantly associated with all discharge outcomes, including FMA-U/L (*ρ* = 0.41, *p* < 0.01), BBT (*ρ* = 0.29, *p* < 0.01), FIM (*r* = 0.42, *p* < 0.01), and K-MBI (*ρ* = 0.40, *p* < 0.01).

**Table II:** Correlation between admission SEP/MEP status and discharge functional scores (*n =* 111)

Factor	Upper limb SEP	Upper limb MEP
FMA-U/L (*n =* 110)	0.41^a^**	0.70^a^**
BBT (*n =* 74)	0.29^a^**	0.40^a^**
FIM (*n =* 111)	0.42^b^**	0.39^b^**
K-MBI (*n =* 111)	0.40^a^**	0.53^a^**

SEP: somatosensory evoked potentials; MEP: motor evoked potentials; FMA-U/L: Fugl-Meyer Assessment for upper limb; BBT: Box and Block Test; FIM: Functional Independence Measure; K-MBI: Korean version of the modified Barthel Index.

a Spearman’s ρ;

b Pearson’s r.

** *p <* 0.01.

Upper limb MEP status also demonstrated significant positive correlations with all outcome measures. The strongest association was observed with FMA-U/L (*ρ* = 0.70, *p* < 0.01), followed by K-MBI (*ρ* = 0.53, *p* < 0.01), BBT (*ρ* = 0.40, *p* < 0.01), and FIM (*r* = 0.39, *p* < 0.01).

### Predictive value of admission SEP status for functional recovery on discharge: mixed-effects model results

To evaluate the predictive value of upper limb SEP status on admission for post-rehabilitation functional outcomes, a series of linear mixed-effects models were constructed. In these models, SEP status (non-responsive, abnormal, or normal) was included as a fixed effect, while individual patient ID was entered as a random effect to account for within-subject variability. Covariates included admission duration and baseline functional scores.

Compared with the SEP non-responsive group, the SEP abnormal group exhibited greater improve-ments in upper limb motor function (FMA-U/L), although this difference did not reach statistical significance (*B* = 4.51, *p* = 0.087). A similar trend was observed in the SEP normal group (*B* = 5.28, *p* = 0.067), accompanied by a small effect size (Cohen’s *f*^2^ = 0.023). For manual dexterity, as measured by the BBT, no significant between-group differences were identified.

In terms of ADL, the SEP normal group showed significantly greater gains compared to the non-responsive group in both FIM (*B* = 10.06, *p* = 0.024, *f*^2^ = 0.040) and K-MBI scores (*B* = 12.36, *p* = 0.025, *f*^2^ = 0.039). Although the SEP abnormal group demonstrated a numerical improvement, the results were not statistically significant. Across all models, baseline functional scores consistently emerged as significant predictors of discharge outcomes (*p* < 0.001).

**Fig. 3:**
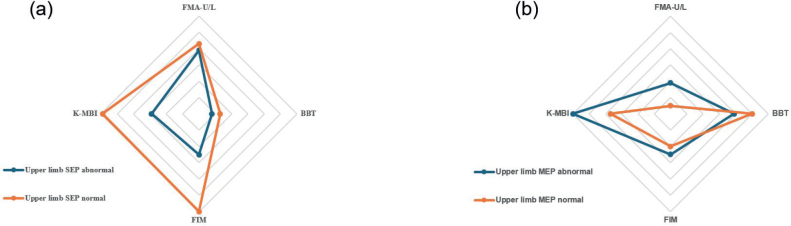
Radar charts illustrating the effect sizes (Cohen’s *f*^2^) from mixedeffects models for discharge functional outcomes. (A) Effect sizes for upper limb SEP status (abnormal and normal) across FMAU/L, BBT, FIM, and KMBI. (B) Effect sizes for upper limb MEP status (abnormal and normal) across the same outcomes. SEP: somatosensory evoked potentials; MEP: motor evoked potentials; FMA-U/L: Fugl-Meyer Assessment for upper limb; BBT: Box and Block Test; FIM: Functional Independence Measure; K-MBI: Korean version of the modified Barthel Index. According to Cohen (29), *f*^2^ values of 0.02, 0.15, and 0.35 represent small, medium, and large effects, respectively.

Detailed regression coefficients are presented in Table III, and corresponding effect sizes for each outcome are visualized in Fig. 3.

**Table III:** Mixed-effects model estimating the predictive value of admission SEP status for discharge functional outcomes (*n =* 111)

Independent variable	Estimate (*B*)	SE	*p*-value	CI	Cohen’s *f*^2^
Mixed-effects model for FMA-U/L (*n =* 110)					
SEP abnormal	4.51	2.61	0.087	–0.67–9.70	0.019
SEP normal	5.28	2.85	0.067	–0.38–10.94	0.023
Admission duration	0.13	0.09	0.185	–0.06–0.31	
Baseline score	0.84	0.04	< 0.001**	0.76–0.92	
Constant term	4.34				
Mixed effects model for BBT (*n =* 74)					
SEP abnormal	1.73	5.19	0.740	–8.63–12.09	–0.012
SEP normal	3.61	5.23	0.492	–6.83–14.05	–0.007
Admission duration	0.27	0.14	0.067	–0.02–0.55	
Baseline score	0.95	0.07	0.001**	0.80–1.10	
Constant term	5.42				
Mixed effects model for FIM (*n =* 111)					
SEP abnormal	5.01	4.04	0.217	–2.99–13.01	0.005
SEP normal	10.06	4.40	0.024*	1.35–18.79	0.040
Admission duration	0.28	0.14	0.048*	0.00–0.57	
Baseline score	0.91	0.06	< 0.001**	0.80–1.02	
Constant term	6.16				
Mixed effects model for K-MBI (*n =* 111)					
SEP abnormal	7.03	4.98	0.161	–2.85–16.92	0.009
SEP normal	12.36	5.45	0.025*	1.55–23.17	0.039
Admission duration	0.15	0.18	0.393	–0.19–0.50	
Baseline score	0.75	0.06	< 0.001**	0.62–0.87	
Constant term	18.17				

According to Cohen (29), *f*^2^ values of 0.02, 0.15, and 0.35 represent small, medium, and large effects, respectively.

SEP: somatosensory evoked potentials; FMA-U/L: Fugl-Meyer Assessment for upper limb; BBT: Box and Block Test; FIM: Functional Independence Measure; K-MBI: Korean version of the modified Barthel Index.

* *p <* 0.05,

** *p <* 0.01, statistically significant difference.

### Predictive value of admission MEP status for functional recovery on discharge: mixed-effects model results

To assess the predictive value of upper limb MEP status on admission for functional recovery on discharge, a series of linear mixed-effects models were constructed. In each model, MEP status (non-responsive, abnormal, or normal) was included as a fixed effect, and patient ID was modelled as a random effect to account for within-subject variability. Admission duration and baseline functional scores were entered as covariates.

In the analysis of upper limb motor recovery (FMA-U/L), the MEP abnormal group (*B* = 4.63, *p* = 0.086) and MEP normal group (*B* = 4.12, *p* = 0.220) both demonstrated greater improvement compared with the non-responsive group, although these differences were not statistically significant. The corresponding effect sizes were small (*f*^2^ = 0.005–0.019).

For manual dexterity as measured by the BBT, the MEP normal group showed a significantly greater improvement than the non-responsive group (*B* = 10.81, *p* = 0.039), with a small-to-moderate effect size (*f*^2^ = 0.050). The MEP abnormal group exhibited a similar trend (*B* = 9.44, *p* = 0.059), though this result did not reach statistical significance (*f*^2^ = 0.039).

With regard to ADL, both the MEP abnormal group (*B* = 11.12, *p* = 0.008, (*f*^2^ = 0.060) and the MEP normal group (*B* = 10.75, *p* = 0.028, *f*^2^ = 0.037) demonstrated significantly greater improvements in K-MBI scores compared with the non-responsive group. In contrast, no statistically significant group differences were observed for FIM scores; however, the MEP abnormal group showed a trend towards greater improvement (*B* = 6.47, *p* = 0.058).

Across all models, baseline functional scores emerged as consistent and significant predictors of discharge outcomes (*p* < 0.001). Additionally, admission duration was a significant covariate in the models predicting BBT and FIM outcomes.

Detailed regression results are provided in Table IV, and the corresponding effect sizes for each functional domain are illustrated in Fig. 3.

**Table IV:** Mixed-effects model estimating the predictive value of admission MEP status for discharge functional outcomes (*n =* 111)

Independent variable	Estimate (*B*)	SE	*p*-value	CI	Cohen’s *f*^2^
Mixed-effects model for FMA-U/L (*n =* 110)					
MEP abnormal	4.63	2.67	0.086	–0.67–9.94	0.019
MEP normal	4.12	3.33	0.220	–2.49–10.73	0.005
Admission duration	0.12	0.10	0.194	–0.06–0.31	
Baseline score	0.81	0.05	< 0.001**	0.70–0.92	
Constant term	6.86				
Mixed-effects model for BBT (*n =* 74)					
MEP abnormal	9.44	4.92	0.059	–0.38–19.26	0.039
MEP normal	10.81	5.15	0.039*	0.54–21.08	0.050
Admission duration	0.37	0.15	0.015*	0.07–0.66	
Baseline score	0.94	0.07	< 0.001**	0.80–1.08	
Constant term	–3.32				
Mixed-effects model for FIM (*n =* 111)					
MEP abnormal	6.47	3.37	0.058	–0.21–13.16	0.025
MEP normal	6.83	3.88	0.082	–0.86–14.53	0.020
Admission duration	0.34	0.15	0.025*	0.04–0.64	
Baseline score	0.92	0.06	< 0.001**	0.81–1.03	
Constant term	5.64				
Mixed-effects model for K-MBI (*n =* 111)					
MEP abnormal	11.12	4.09	0.008**	3.01–19.23	0.060
MEP normal	10.75	4.82	0.028*	1.19–20.30	0.037
Admission duration	0.24	0.18	0.184	–0.12–0.60	
Baseline score	0.74	0.06	< 0.001**	0.62–0.86	
Constant term	16.74				

According to Cohen (29), *f*^2^ values of 0.02, 0.15, and 0.35 represent small, medium, and large effects, respectively.

MEP: motor evoked potentials; FMA-U/L: Fugl-Meyer Assessment for upper limb; BBT: Box and Block Test; FIM: Functional Independence Measure; K-MBI: Korean version of the modified Barthel Index.

* *p <* 0.05,

** *p <* 0.01, statistically significant difference.

## DISCUSSION

This study evaluated the prognostic value of upper limb SEP and MEP measured on admission for functional outcomes on discharge in patients with subacute stroke. The results demonstrated that patients with preserved SEP responses, particularly those classified as normal, achieved significantly greater gains in ADL as measured by both the FIM and the K-MBI compared with the non-responsive group. In contrast, SEP status was associated only with a non-significant trend towards improvement in upper limb motor function (FMA-U/L) and showed no significant difference in manual dexterity (BBT). Regarding MEP, significant improvements were observed in BBT performance for the normal group and in K-MBI scores for both the abnormal and normal groups, whereas the FIM showed only a near-significant trend in the abnormal group.

The SEP findings align with previous reports indicating that the integrity of the dorsal column–medial lemniscal pathway is associated with superior functional recovery after stroke (24, 25). Preservation of the sensory pathway may facilitate the integration of proprioceptive feedback during task-specific rehabilitation, thereby enhancing ADL performance even in the absence of substantial motor improvement (13). The stronger association with ADL than with direct motor gains suggests that intact sensory function may support compensatory strategies that increase overall independence rather than solely promoting motor restitution.

The MEP results are consistent with earlier studies demonstrating that corticospinal tract integrity is a critical determinant of precise motor control and hand dexterity in stroke rehabilitation (8, 26). The significant association between MEP status and BBT performance underscores the role of preserved motor pathway conduction in enabling repetitive and coordinated upper limb movements. The finding that MEP status predicted improvement in K-MBI but not in FIM may be attributable to differences in the measurement focus of the 2 ADL instruments. The K-MBI primarily assesses physical and motor-based tasks, which are more directly influenced by upper limb motor capacity, whereas the FIM also incorporates cognitive and communication components that may attenuate the measurable impact of motor pathway integrity on the total score (27, 28).

Taken together, the present findings indicate that admission SEP and MEP status are associated with discharge functional outcomes in patients with subacute stroke, independent of baseline functional performance and admission duration. Although several associations reached statistical significance, the corresponding effect sizes were generally small to moderate. This suggests that SEP and MEP status provide meaningful but incremental prognostic information beyond baseline functional status, rather than serving as dominant determinants of recovery.

The distinct predictive patterns of SEP and MEP observed in this study suggest that these measures serve complementary roles in functional recovery assessment. SEP appears to be more closely related to overall independence in daily living, likely through its contribution to movement precision and safety, while MEP is more strongly associated with fine motor skills and selected ADL components. These findings support the combined use of SEP and MEP in early rehabilitation evaluations to obtain a more comprehensive prognostic profile in patients with subacute stroke.

Several limitations should be considered. First, the retrospective design restricts causal interpretation. Second, although SEP and MEP were analysed separately, their combined predictive value and potential interaction effects were not assessed. Third, other potentially influential factors such as lesion location, stroke severity, and rehabilitation intensity were not fully controlled, which may have influenced the results. Future prospective research should incorporate multimodal neurophysiological and clinical variables into predictive models to improve prognostic accuracy.

In conclusion, SEP and MEP status on admission offer valuable prognostic insight into upper limb motor performance and ADL outcomes on discharge in patients with subacute stroke. Incorporating these neurophysiological assessments into early rehabilitation planning can facilitate individualized treatment strategies aimed at maximizing functional recovery.
